# Optical coherence tomography angiography in dural carotid-cavernous sinus fistula

**DOI:** 10.1186/s12886-016-0278-1

**Published:** 2016-07-07

**Authors:** Marcus Ang, Chelvin Sng, Dan Milea

**Affiliations:** Singapore National Eye Center, Singapore Eye Research Institute, 11 Third Hospital Avenue, Singapore, 168751 Singapore; Ophthalmology and Visual Sciences Department, Duke-NUS, Singapore, Singapore; National University Health System, Singapore, Singapore

**Keywords:** Optical coherence tomography, Angiography, Carotid-cavernous sinus fistula

## Abstract

**Background:**

Recently, applications of optical coherence tomography angiography (OCTA) have been limited to the retina and posterior segment. Although early studies have described its use for other clinical applications, its role in anterior segment vasculature and optic disc imaging has been limited thus far.

**Case presentation:**

We describe a novel clinical application of OCTA in a patient with dural carotid-cavernous sinus fistula (CCF), which was complicated by increased intra-ocular pressure (IOP). In this case report, we used the OCTA to delineate increased epsicleral venous flow in the affected eye with secondary raised IOP. Current measurements of episcleral venous pressure are either invasive or provide highly variable results, thus the OCTA may have the potential to provide a more reliable approach to assess episcleral vasculature. We also describe the use of OCTA to detect early glaucomatous nerve damage, associated with focal reductions in peripapillary retinal perfusion.

**Conclusions:**

We present an early report of using OCTA of the anterior segment to allow rapid, non-invasive delineation of abnormal episcleral venous plexus secondary to dural CCF. The OCTA was also useful for detecting early reduction in peripapillary retinal perfusion, which suggests early glaucomatous optic neuropathy. This suggests that OCTA may have a role for determining risk of glaucoma in patients with CCF in future.

## Background

Anterior segment angiography has a variety of clinical applications, which may range from evaluation of corneal vascularization [1], to scleral inflammatory disorders [2, 3]. Optical coherence tomography angiography (OCTA) has been described for the delineation of vessels in the optic disc, retina and most recently, the anterior segment [4]. These non-contact imaging systems detect phase variations or changes in reflectivity to detect vascular flow, with the added benefit of concurrently obtaining optical coherence tomography (OCT) scans of the surrounding tissue [5].

Since current OCTA systems are optimized for the retina and optic disc, we had previously described a technique adapted to perform scans in the anterior segment for normal corneal and limbal vessels [6]. Carotid-cavernous sinus fistulas (CCF) result from abnormal connections between the carotid arterial system and the cavernous sinus, leading to ophthalmic complications due to arterialization of the ocular venous system [7]. In this case report, we describe the use of OCTA to delineate the episcleral venous plexus, which may be the primary mechanism for secondary glaucoma in CCF.

## Case presentation

**Fig. 1:**
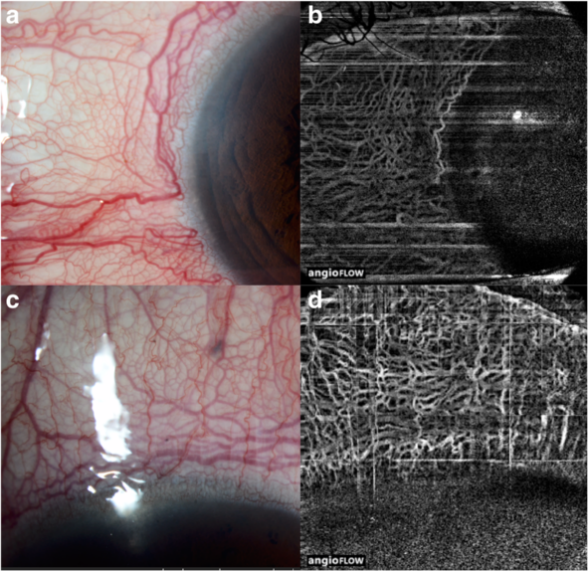
Slit-lamp microscopy revealed dilated and tortuous conjunctival vessels in all four limbal quadrants of the eye, especially in the nasal (**a**) and superior quadrants (**c**) in the left eye of the patient with the dural carotid-cavernous fistula. Optical coherence tomography angiography allows for delineation of the deeper dilated episceral veins in the corresponding quadrants (**b & d**). The aquous humour enters the Schlemm canal through the trabecular meshwork and intrascleral emissary channels to empty into the episcleral venous plexus

**Fig. 2:**
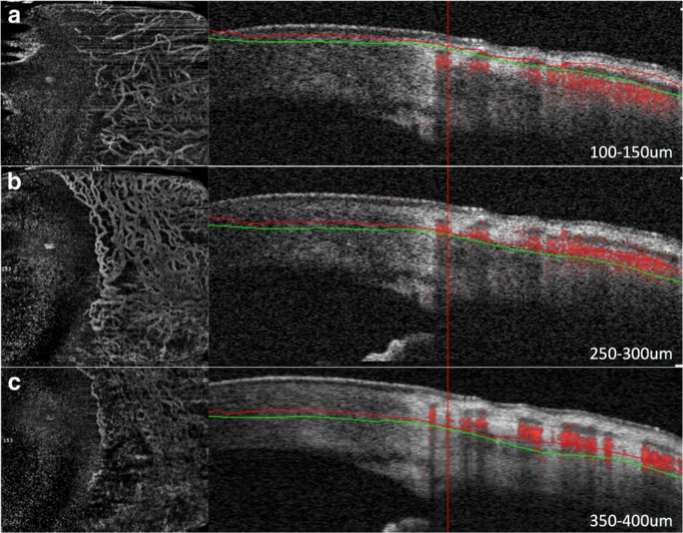
Optical coherence tomography angiography disclosing increased blood flow in the congested episcleral venous network in the temporal quadrant of the eye. Although the superficial conjunctival vessels are mildly dilated (**a**), most of the increased flow is detected on the B-scan (indicated by the intense red areas on the B-scan, right) in the episcleral venous plexus (**b**). There are virtually no dilated vessels in the deeper scleral areas (**c**)

**Fig. 3:**
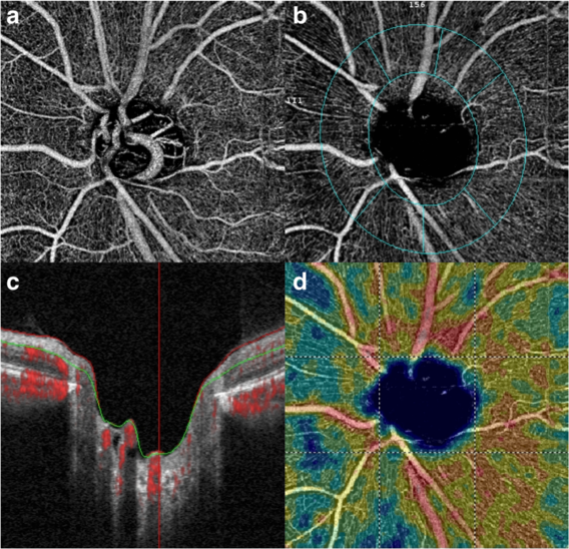
Optical coherence tomography angiography of the left optic disc, suggesting early glaucomatous optic nerve damage (**a**). The in-built software allows analysis of the peripapillary vascular flow and nerve fibre layer (**b**) suggesting neuroretinal rim thinning(**c**) and reduction in peripapillary retinal perfusion (**d**)

We present a case of a 54-year-old Chinese man with a history of treated hypertension and diabetes mellitus, referred for suspected spontaneous left dural CCF. The patient complained of a chronically red left eye and double vision in the left gaze. Initial examination disclosed best corrected visual acuity 20/20 in the right eye and 20/25 in the left eye, associated with a left relative afferent pupillary defect and severely reduced color vision on Ishihara testing in his left eye only. Humphrey visual field assessment disclosed diffuse defects in the left eye, but was normal in the right eye. Slit lamp examination disclosed a red left eye with dilated corkscrew vessels, but no evidence of angle closure, blood in the Schlemm canal, or other causes of raised intraocular pressure. Intraocular pressure (IOP) was 28 mmHg in the left eye, and 14 mmHg in the right, healthy eye. Fundoscopy disclosed dilated retinal veins on the left side, but no venous stasis retinopathy, or choroidal detachment. The remainder of the ophthalmic examination disclosed mild left abduction deficit, mild ptosis and 3 mm proptosis on the left side. Partial embolization of the angiographically confirmed left dural CCF resulted in incomplete clinical recovery: despite complete regression of the left optic neuropathy and of the left abduction deficit, the left eye remained red, associated with raised intraocular pressure requiring topical medication. Optical coherence tomography angiography of the anterior segment was performed, using a described technique [6], with optic disc imaging as well using the AngioVue (Optovue Inc. Fremont, CA, USA). Our study followed the principles of the Declaration of Helsinki, with informed consent obtained and ethics approval obtained from our local Institutional Review Board (CIRB Ref no: 2015/3078). OCTA showed engorged episcleral vessels, which were better delineated than using clinical evaluation alone (Fig. 1). The OCTA was able to analyze the location and depth of the tortuous, abnormal vessels, as well as the increased flow detected on the B-scan cross-sectional OCTA scans (Fig. 2). Posterior pole OCTA of the affected eye disclosed glaucomatous nerve damage and reduction in the peripapillary flow (Fig. 3) [8].

## Conclusions

The episcleral venous outflow is a key component of intraocular pressure, as embodied by the modified Goldmann equation, which can be thought of as the pressure required to move aqueous humor out of the eye through the resistance of the trabecular meshwork and the pressure in the episcleral veins [9]. Optical coherence tomography angiography has been recently described for the delineation of blood flow within vessels in the optic disc, retina as well as a novel application in the anterior segment [10]. Here, we used a novel OCTA technique, dedicated to the anterior segment, to explore a patient with a dural sinus CCF, due to abnormal connections between the carotid arterial system and the cavernous sinus [7].

One of the main presumed mechanisms for raised IOP associated with CCF is increased episcleral venous pressure [7]. Objective measurements of episcleral venous pressure are either invasive or provide highly variable results [9]. The OCTA may be a more reliable approach to assess episcleral vasculature. In addition, we have also used OCTA to image the optic disc to detect early glaucomatous nerve damage, which has been associated with reduced peripapillary retinal perfusion in focal areas around the disc [8]. The potential clinical applications of OCTA for the anterior segment, apart from those already mentioned, could also extend to assessment of graft vascularization and inflammation [11], studying limbal vasculature associated with limbal stem cell deficiency or allergic eye disease [12], or even evaluation of bleb vascularity and morphology after glaucoma surgery [13].

In summary, this early clinical report describes, for the first time, the OCTA features in dural CCF, suggesting that OCTA allows rapid, non-invasive, in-depth visualization of abnormal episcleral vasculature in CCF. Further quantitative studies are needed to assess the link between abnormal episcleral vasculature and the risk for glaucoma in cases of dural CCF.

## Abbreviations

OCTA, optical coherence tomography angiography; OCT, optical coherence tomography; CCF, carotid-cavernous sinus fistula; IOP, intraocular pressure
